# Surfactant-mediated extraction of capsaicin from *Capsicum annuum* L. fruit in various solvents

**DOI:** 10.1016/j.heliyon.2022.e10273

**Published:** 2022-08-17

**Authors:** Muhammad Waqas, Dildar Ahmed, Muhammad Tariq Qamar

**Affiliations:** Department of Chemistry, Forman Christian College (A Chartered University), Lahore, Pakistan

**Keywords:** *Capsicum annuum*, Capsaicin, Extraction, Surfactant-assisted, Solvent effect

## Abstract

Capsaicin is a valuable compound found in *Capsicum annuum*. The present study aimed to explore the efficiency of different solvents and surfactants on its extraction by maceration. Ethyl acetate was found to be the best solvent followed by dichloromethane and acetone, respectively. Overall order of efficiency of the solvents used was this: ethyl acetate > dichloromethane > acetone > glycerol > acetonitrile > methanol > acetic acid > toluene. Extractability of ethyl acetate for capsaicin remained unaffected by the surfactants. Tween-80 had very positive effect on the extraction efficiency of dichloromethane (DCM) and acetone. Kinetics of the extraction with the most efficient solvent ethyl acetate showed extraction of capsaicin to follow a pseudo-second order kinetic model. In conclusion, for extraction of capsaicin from green chili, ethyl acetate was the most powerful amongst the solvents used in the present work and tween-80 had a notable positive effect on the efficiency of DCM and acetone.

## 1 Introduction

*Capsicum annuum* L. or chili is a well-known plant found through the world. Its fruit is commonly used as a spice and added in various types of cuisines and is also used in traditional medicine. The fruit of *Capsicum* species or hot peppers contains a group of compounds that give them their characteristic pungent flavour [1]. These compounds are called capsaicinoids. The major component of this group is capsaicin. Capsaicin and its related compounds are used in folk medicine for different purposes, such as to relieve pain and to treat arthritis [2, 3, 4]. The structures of some capsaicinoids are given in Table 1.

**Table 1:** Capsaicin and related compounds (capsaicinoids).

Capsaicinoids names	Structures
Capsaicin	
Dihydrocapsaicin	
Nordihydrocapsaicin	
Homodihydrocapsaicin	
Homocapsaicin	
Nonivamide	

Chemically capsaicin is 8-methyl-*N*-vanillyl-6-nonenamide. In its pure form, it is a colorless, crystalline compound. It is hydrophobic and insoluble in water. The compound has been found to have remarkable property to relieve pain [5]. Apart from its use in food and medicine, capsaicin is also used in cosmetics [6]. In order to explore its possible applications to treat chronic diseases, the compound is also used in research in various fields of medicine, such as cancer [7, 8, 9]. The literature shows numerous studies on the medicinal activities of capsaicin and related compounds [10, 11, 12, 13]. Its analgesic and painkilling effect is well-known which has led to its use in pain relieving creams. Studies have also highlighted its role in treating metabolic disorders such as those related to obesity and diabetes. It has also found effective in treating some types of human cancers such as those affecting lungs, stomach, colon, and breast [13]. In fact, the literature shows an increasing number of studies focusing on the anti-cancer activities of capsaicin that has increased the value of this compound up to manifold [14, 15].

Generally, capsaicinoids obtained from hot red chilis contain 69% of capsaicin, 22% dihydrocapsaicin, 7% nordihydrocapsaicin, 1% homocapsaicin and 1% homodihydracapsaicin [16, 17, 18].

In view of their importance as therapeutic agents, numerous studies report isolation and identification of capsaicinoids from chili and their applications [16, 19, 20]. For extraction of these compounds, several solvents and techniques have been evaluated.

In the recent years, surfactants-assisted extraction of bioactive compounds has gain attention [21, 22, 23, 24]. Surfactants are amphiphilic compounds and have a hydrophilic and a lipophilic part. In aqueous media, the surfactant molecules form micelles when the concentration of the surfactant is higher than its CMC (critical micelle concentration). They form micelles by orienting themselves in a way that their hydrophobic parts make the inner core while the hydrophilic parts make the micellar surface. Surfactants assist the extraction of bioactive compounds from the plant cells by playing role in cleaving the cell membrane and cell wall and by increasing the solubility of otherwise less soluble compounds in aqueous medium [25]. The factors affecting their effectiveness for extraction of chemical compounds from biomasses include nature of surfactants depending upon the nature of target molecules. For this purpose, the surfactants commonly used include cetyltrimethylammonium bromide (CTAB), sodium dodecyl sulfate (SDS) and tween-80.

There are many organic liquids that are used as solvents to extract chemical compounds from plants and other organisms. For extraction of capsaicin from chili, several studies have been conducted using various solvents. These include methanol-water, methanol-surfactant, methanol-water-surfactant, and water-surfactant solutions [26].

Quantification of capsaicin in the extracts were determined by UV-visible spectrophotometry method. This method has an obvious limitation since the extracts not only contained the desired compound capsaicin but also other phytochemicals extractable under the experimental conditions, which may contribute to the absorbance. However, the method is widely used as it is robust, convenient, and cost-effective and gives a reasonable estimate of the desired compound. The method has also been used for the quantification of capsaicin [19, 27].

With this background in view, the current work was designed with the objectives to evaluate the effectiveness of different solvents for extraction of capsaicin from chili and to investigate the effect of anionic, cationic and non-ionic surfactants on the extraction efficiency of the solvents with significant effectiveness.

## 2 Materials and methods

### 2.1 Materials and reagents

The chemicals used in the current study were of analytical grade. CTAB, SDS, tween-80 was purchased from Sigma-Aldrich Chemicals (St. Louis, US). The solvents methanol, ethyl acetate and acetone, toluene, glycerol, acetonitrile, acetic acid and dichloromethane were obtained from Merck (Darmstadt, Germany). Standard capsaicin was purchased from Chem-Impex Int'l Inc. (Chicago, US).

### 2.2 Collection of plant material

A sample of fresh chili (*Capsicum annuum* L.) fruit was purchased from a local market of Lahore, Pakistan. The fruit was green. It was dried in shade for 10 days at room temperature and ground to obtain a fine powder. This powder was used for extraction studies.

### 2.3 Extraction with different solvents

Several solvents were used for extraction, which included methanol, ethyl acetate, dichloromethane, acetic acid, toluene, glycerol, acetonitrile, and acetone. Based on the preliminary runs, a measured amount of the chili powder (1 g) was soaked in 50 mL of each solvent and the mixture was put on shaking for 6 h on an orbital shaker at room temperature. The mixture was filtered under gravity using Whatman filter paper no 1. In order to make it appropriate for UV-Visible spectrophotometric analysis, the filtrate was diluted with the same solvent (1 mL of the filtrate was diluted with 9 mL of the solvent). Each experiment was carried out three times.

### 2.4 Calculation of yield

The extraction yield of capsaicin was calculated by UV-visible spectrophotometry method according to the Lambert-Beer law. A standard curve of the standard capsaicin was drawn by measuring the absorbance of dilutions at 280 nm (the λ_max_ of capsaicin) and molar absorptivity was calculated. Absorbance of each extract was then recorded at 280 nm. The value of molar absorptivity was used to calculate the yield of capsaicin in the extract using equation of Lambert-Beer law.

### 2.5 Extraction in the presence of surfactants

Three most effective solvents in the present study were ethyl acetate, dichloromethane, and acetone. Effect of surfactants on their extractability was evaluated. Three different surfactants were used (a) anionic (sodium dodecylsulfate or SDS), (b) non-ionic (tween-80) and (c) cationic (cetrimonium bromide or CTAB). A known amount of the chili powder (1 g) was mixed in a given amount (30 mL) of a given solvent and extraction was carried out in the presence or absence of a surfactant. The amount of a given surfactant added was a little more than its CMC. A summary of the parameters used are given in Table 2. Each experiment was carried out at least in triplicate.

**Table 2:** A summary of the parameters used for surfactant-assisted extraction of capsaicin from ground dried fresh green fruit of *Capsicum annuum*

Solvent	Solvent to solid ratio (mL/g)	SDS (g)	CTAB (g)	Tween-80 (mL)
(1) Acetone	30	0.714	0.086	0.21
(2) Ethyl acetate				
(3) Dichloromethane				

The absorbance was measured at 280 nm to calculate the yield of capsaicin using Lambert-Beer law; shaking time: 6 h.

### 2.6 Study of kinetics of capsaicin extraction

A known amount of ground dried fresh chili powder (0.1 g) was soaked in 10 mL ethyl acetate solvent and was allowed to shake for 10, 20, 30, 60, 120, 240, 360, 480 and 1440 min on a shaker. The mixture was filtered under gravity using Whatman filter paper no 1. Then, 1 mL of the filtrate was diluted with 3 mL methanol for the measurement of absorbance at 280 nm. Extraction yield of capsaicin was calculated using Lambert-Beer law. Each experiment was carried out in triplicate.

### 2.7 Statistical analysis

All the determinations were made at least three times, and statistical mean was calculated with standard deviation using Microsoft Excel 365 (Las Vegas, NV, US).

## 3 Results and discussion

Capsaicin, the pungent principle in chili, is a valuable natural product because of having medicinal and analytical applications. It is highly valuable to extract this bioactive substance in high yield. Although the literature reports several studies to this effect, the yield is still a problem. The present study, therefore, was planned to explore methods to extract capsaicin from *C. annuum* in high yield. The objective was to assess the effect of three different surfactants on the extraction of capsaicin using a variety of solvents. The surfactants used included SDS, CTAB, and Tween-80. The SDS (sodium dodecylsulfate) is an anionic, CTAB (cetyltrimethylammonium bromide) is cationic while Tween-80 is a neutral surfactant.

### 3.1 Extraction of capsaicin in different solvents

Capsaicin was extracted from dried powdered green chili using different solvents and in the presence of three different surfactants. The quantification of capsaicin in the extracts were done according to UV-visible spectrophotometry method by measuring absorbance at the λmax of capsaicin. The results are shown in Table 3.

**Table 3:** Extraction yield of capsaicin from fresh green chili powder in different solvents.

Solvent	Ethyl acetate	DCM	Acetone	Glycerol	Acetonitrile	Methanol	Acetic acid	Toluene
**Yield** (mg/10 g)	73.97 ± 2.25	35.98 ± 4.36	28.8 ± 2.92	17.78 ± 0.20	7.87 ± 1.82	7.37 ± 3.24	4.08 ± 0.94	2.77 ± 2.03

As Table 3 shows, ethyl acetate was found to be the most effective solvent to extract capsaicin from the powder of dried green chili followed by dichloromethane and acetone, respectively. Ethyl acetate provided maximum yield (73.97 mg out of 10 g sample). On the other hand, dichloromethane provided 35.98 mg out of 10 g sample. Acetone also proved to be a good solvent after dichloromethane and provided 28.88 mg out of 10 g sample. The extraction efficiency of ethyl acetate was, therefore, remarkably higher than the other solvents used in the study. Capsaicin and other capsaicinoids are phenolic compounds and the efficiency of ethyl acetate to extract phenolic has been reported in many studies [28]. The importance of the study stems from the need to discover the most efficient ways to extract capsaicin and related compounds as well as the other bioactive compounds found in chili such as phenolics and carotenoids [29, 30]. Since one of the most important factors affecting extraction of chemical constituents of a plant material is choice of solvents, the findings of the present work are important for future studies. Ethyl acetate has been found not only a very efficient extracting medium for capsaicin, but it is also considered an environmentally friendly or green solvent [31, 32, 33]. The efficacy of ethyl acetate in extracting phenolics has been attributed to its moderately polar nature [34]. An ethyl acetate extract of chili, therefore, should be considered as safe product for industrial applications such as its use in pain killer creams for topical applications [35, 36].

### 3.2 Effect of surfactants on the extractability of solvents

Effect of surfactants on the extraction of capsaicin from chili material was investigated and the results are shown in Table 4.

**Table 4:** Effect of surfactants on extraction of capsaicin from fresh green chili fruit powder in different solvents.

Solvent and Surfactant	Yield in different solvents (mg/10 g)		
	Ethyl acetate	Dichloromethane	Acetone
Solvent	73.97 ± 2.255	35.98 ± 4.362	28.8 ± 2.916
Solvent + SDS	72.29 ± 2.941	46.53 ± 11.079	43.7 ± 6.689
Solvent + CTAB	72.62 ± 2.649	35.57 ± 1.302	46.7 ± 13.837
Solvent + Tween-80	72.74 ± 0.641	71.20 ± 1.273	61.6 ± 4.348

As Table 4 shows, the surfactants had little effect on the extraction efficiency of ethyl acetate. However, all the surfactants had a pronounced impact on the efficiency of acetone to extract capsaicin, and Tween-80 was most effective followed by CTAB. Tween-80 had very positive effect on the extraction efficiency of dichloromethane, the trend supported other studies that have shown non-ionic surfactants to be more effective than the ionic ones [23]. SDS too had a fairly good positive effect. However, CTAB had almost no effect. Since Tween-80 is known to be a nontoxic surface acting compound, it can be used for extracting valuable bioactive compounds for nutraceutical or therapeutic purposes without any concern about the safety [21]. The efficacy of tween-80 in extracting phenolic compounds has also been demonstrated by other studies [37]. It has been suggested that the surfactant binds itself with phenolics through hydrogen bonding and thereby assisted in their extraction [38].

### 3.3 Study of rate kinetics for capsaicin extraction

**Figure 1:**
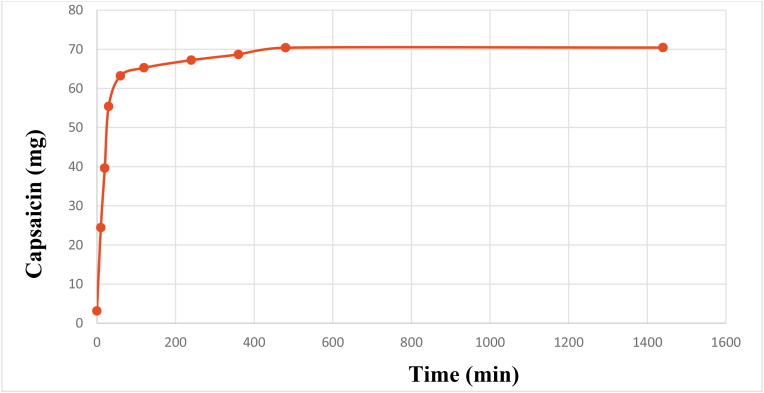
Amount of capsaicin extract from chili in ethyl acetate solvent as a function of time; each value is a mean of three experiments as quantified by measuring absorbance of the extracts at 280 nm.

Amount of capsaicin extract from chili as a function of time was determined for kinetics study. The results are shown in Figure 1.

The kinetic study of the extraction with the most efficient solvent ethyl acetate exhibited interesting results, according to which the rate of extraction of capsaicin with ethyl acetate appeared to follow the second order kinetic model. Similar trend has been found in extraction of polyphenols in other studies [39, 40]. Notably, during the first hour of the extraction, there was a rapid extraction of capsaicin after the extraction slowed down and attained a steady state. The study, therefore, suggests that most capsaicin is extractable in the first 60 min of the process.

As capsaicin is a valuable bioactive compound with various actual and potential medicinal applications, exploring efficient methods for its extraction from chili is important [13, 29]. Numerous studies have been carried out to this end, but the extraction yield is still a problem [36]. The present work, therefore, is a contribution to the ongoing work on this subject paving way for further in-depth studies. The efficacy of ethyl acetate as extracting medium for capsaicin has been demonstrated with the finding that most of extraction occurs within the first hour of the process.

## 4 Conclusions

Capsaicin is a highly valuable compound found in chili peppers. It is, therefore, of great importance to discover efficient methods of its extraction. The realization led to the current study that demonstrated ethyl acetate to be a highly effective solvent for extracting capsaicin from chili. Out of the several solvents used, ethyl acetate gave the highest extraction yield with the efficiency order: ethyl acetate > dichloromethane > acetone > glycerol > acetonitrile > methanol > acetic acid > toluene. Moreover, the non-ionic surfactant tween-80 strongly enhanced the extracting ability of acetone and dichloromethane. Thus, based on the findings of the present study, new methods may be developed and optimized to obtain high yield of capsaicin from chili.

## Declarations

### Author contribution statement

Muhammad Waqas: Performed the experiments; Analyzed and interpreted the data; Wrote the paper. Dildar Ahmed: Conceived and designed the experiments; Analyzed and interpreted the data; Wrote the paper. Muhammad Tariq Qamar: Analyzed and interpreted the data; Contributed reagents, materials, analysis tools or data; Wrote the paper.

### Funding statement

This research did not receive any specific grant from funding agencies in the public, commercial, or not-for-profit sectors.

### Data availability statement

Data will be made available on request.

### Declaration of interest statement

The authors declare no conflict of interest.

### Additional information

No additional information is available for this paper.
